# The sterility and antimicrobial potential of 3M™ Micropore™ tape in a lower- to middle-income country

**DOI:** 10.4102/jcmsa.v4i1.290

**Published:** 2026-02-13

**Authors:** Innocent Mukama, Mahendra Daya, Khine Swe Swe/Han, Yesholata Mahabeer

**Affiliations:** 1 Faculty of Health Sciences, University of KwaZulu-Natal, Durban, South Africa; 2 Department of Plastic and Reconstructive Surgery, Faculty of Health Studies, University of KwaZulu-Natal, Durban, South Africa; 3 Department of Medical Microbiology, Faculty of Health Sciences, University of KwaZulu-Natal, Durban, South Africa; 4 Department of Medical Microbiology, National Health Laboratory Services, Durban, South Africa; 5 Department of Medical Microbiology, Laboratory services, University of KwaZulu-Natal, Durban, South Africa

**Keywords:** tape, sterility, bacterial, fungal, wound

## Abstract

Medical adhesives such as tape are used in wound care to hold down wound dressings onto the skin. Among these, 3M™ Micropore™ tape is widely used; however, limited data are available on its sterility and antibacterial effects, especially in environments where infection control practices may be compromised. We explored the sterility and antibacterial potential of factory-packaged 3M™ Micropore™ surgical tape. This study employed a prospective, experimental laboratory-based research design. A total of 48 separate pieces of tape were assessed. To investigate sterility, a *thioglycollate broth* and *Sabouraud dextrose* with chloramphenicol agar plates were used to detect bacterial and fungal growth, respectively. To assess antimicrobial properties, pieces of tape were placed on Mueller-Hinton agar plates lawned with 0.5 McFarland suspension of *Bacillus subtilis to detect* zones of inhibition around the tape. All samples of the tapes analysed found no evidence of bacterial or fungal growth after appropriate incubation periods. Antibacterial activity was not demonstrated. Evidenced by our results, we can conclude that the tapes are sterile. *In vitro*, the tape did not possess any antimicrobial properties.

**Contribution:**

These results suggest that tape-assisted closure, together with safe tape handling, may be, in the future, an important adjunct in the plastic surgeon’s armamentarium. Especially in low- and middle-income countries (LMICs), where cost and supply constraints may limit access to sterile wound dressings, 3M™ Micropore™ tape may offer a viable alternative when applied under hygienic conditions.

## Introduction

Medical adhesive tapes such as 3M™ Micropore™ are widely used across clinical practice because of their affordability, breathability and hypoallergenic properties.^1,2^ In many lower- to middle-income countries, these tapes may be reused or stored under suboptimal conditions, raising concerns about their sterility and potential to harbour pathogenic microorganisms. Despite their extensive use, limited research has evaluated the microbial safety or possible antimicrobial characteristics of Micropore™ tape, particularly when applied directly to wounds.^3,4^

Our unit has long used taping techniques to support wound healing, consistent with historical applications of microporous surgical tape for closed wounds and tension reduction.^3,4^ This experience informed the development of tape-assisted closure (TAC), based on mechanotransductive principles described in the senior author’s PhD.^4^ Tape-assisted closure promotes gradual wound edge approximation, often avoiding grafts or flaps, but relies on applying non-sterile Micropore™ tape directly to open wounds – necessitating microbiological assessment.

This study therefore aimed to determine whether freshly opened Micropore™ tape contains culturable bacteria or fungi and whether it demonstrates any ex vivo antibacterial activity. Establishing microbial safety is essential for ensuring TAC remains a viable, cost-effective wound management option in resource-constrained settings.

## Methods

The tape is packaged in a rectangular clear plastic core with the 3M™ Micropore™ logo and product details displayed. Only intact, distributor-supplied Micropore tape rolls were included, while any rolls with visibly damaged packaging were excluded.

The study was conducted at the microbiology laboratory in Inkosi Albert Luthuli Central Hospital. Random sampling was applied to three packages of Micropore tape, selecting four rolls from each.

### Sterility testing

Two pieces of 3 cm × 2 cm 3M™ Micropore™ tape were cut from each selected roll within a biosafety cabinet using an aseptic technique, making a total of 24 test samples. These were placed in thioglycollate broth and incubated for 48 h at 35°C – 37°C to observe for turbidity, which would indicate possible microbial growth.

An additional 12 test samples of 3-cm Micropore™ tape were placed onto Sabouraud dextrose with chloramphenicol agar. The plates were incubated in an aerobic incubator at 35°C – 37°C for 3 weeks and examined weekly.

### Antibacterial effect

A 0.5 McFarland suspension of *Bacillus subtilis* American Type Culture Collection 6051 was lawned onto Mueller-Hinton agar plates. Twelve test samples of the 3M™ Micropore™ tape were cut into 3 cm samples, were placed onto the plates and incubated under aerobic conditions at 35°C – 37°C. The next day, the plates were examined for the presence or absence of a zone of inhibition around the Micropore™ tape.

### Ethical considerations

Ethical clearance to conduct this study was obtained from the University of KwaZulu-Natal Biomedical Research Ethics Committee (No. BREC/00005344/2023).

## Results

All 24 tape samples cultured in thioglycollate broth remained clear after 48 h, indicating complete sterility, and all 12 samples tested on Sabouraud agar showed no fungal growth. In the antimicrobial assessment, none of the 12 tape samples produced zones of inhibition against *Bacillus subtilis*, confirming the absence of ex vivo antibacterial effects. Together, these findings indicate that the tape is microbiologically clean at the point of testing, and it does not possess inherent antimicrobial properties.

## Discussion

We showed that freshly opened, factory-sealed 3M™ Micropore™ tape was free of bacterial and fungal contamination and exhibited no inherent antimicrobial activity. The absence of contamination at first opening indicates that the manufacturing and packaging processes do not introduce microbial burden, supporting earlier reports that unopened medical tapes generally remain clean while contamination increases rapidly once packaging is breached or tapes are shared among patients.^1,5^ This finding is particularly relevant in environments where resource limitations constrain access to sterile dressings.

Although sterile products remain the preferred option for wound management, the demonstrated cleanliness of Micropore™ tape at initial use suggests it may serve as a safe alternative in selected situations^6,7^, provided that strict single-patient use and aseptic handling protocols are maintained. Recent literature highlights that adhesive tapes can act as vectors of microbial transfer in clinical and operating room settings when handling practices lapse.^5,8,9^ Evidence also indicates that contamination risk is influenced more by storage practices and operator technique than by sterility labelling alone.^8,9^ This underscores the importance of disciplined tape management to preserve the safety of non-sterile products.

The lack of antimicrobial activity confirms that Micropore™ tape should not be relied upon to suppress microbial proliferation; its role remains mechanical rather than antimicrobial. Nevertheless, the sterility at the point of opening aligns with long-standing clinical experience at Inkosi Albert Luthili Central Hospital (IALCH), where Micropore™ has been used safely for selected incisions. These results also mirror previous studies that reported comparable safety between Micropore™ tape and formal dressings in cosmetic breast surgery.^10,11^

## Conclusion

This study demonstrated that 3M™ Micropore™ surgical tape is microbiologically sterile when unopened and but does not possess inherent antimicrobial properties. However, strict aseptic handling and infection control practices should be strictly adhered to.

This underscores the tape’s potential as a cost-effective and accessible option for wound closure in resource-limited healthcare settings, particularly for reconstructive and tissue-assisted procedures. It offers practical and economic advantages and warrants further research to evaluate its long-term clinical outcomes and inform evidence-based wound management guidelines.
